# Behavior and properties of water in silicate melts under deep mantle conditions

**DOI:** 10.1038/s41598-021-90124-7

**Published:** 2021-05-19

**Authors:** Bijaya B. Karki, Dipta B. Ghosh, Shun-ichiro Karato

**Affiliations:** 1 School of Electrical Engineering and Computer Science, Department of Geology and Geophysics, Center for Computation and Technology, Louisiana State University, Baton Rouge, LA 70803 USA; 2 Dpeartment of Earth and Planetary Sciences, Yale University, New Haven, CT 06520 USA

**Keywords:** Solid Earth sciences, Materials science, Physics

## Abstract

Water (H_2_O) as one of the most abundant fluids present in Earth plays crucial role in the generation and transport of magmas in the interior. Though hydrous silicate melts have been studied extensively, the experimental data are confined to relatively low pressures and the computational results are still rare. Moreover, these studies imply large differences in the way water influences the physical properties of silicate magmas, such as density and electrical conductivity. Here, we investigate the equation of state, speciation, and transport properties of water dissolved in Mg_1−*x*_Fe_*x*_SiO_3_ and Mg_2(1−*x*)_Fe_2*x*_SiO_4_ melts (for *x* = 0 and 0.25) as well as in its bulk (pure) fluid state over the entire mantle pressure regime at 2000–4000 K using first-principles molecular dynamics. The simulation results allow us to constrain the partial molar volume of the water component in melts along with the molar volume of pure water. The predicted volume of silicate melt + water solution is negative at low pressures and becomes almost zero above 15 GPa. Consequently, the hydrous component tends to lower the melt density to similar extent over much of the mantle pressure regime irrespective of composition. Our results also show that hydrogen diffuses fast in silicate melts and enhances the melt electrical conductivity in a way that differs from electrical conduction in the bulk water. The speciation of the water component varies considerably from the bulk water structure as well. Water is dissolved in melts mostly as hydroxyls at low pressure and as –O–H–O–, –O–H–O–H– and other extended species with increasing pressure. On the other hand, the pure water behaves as a molecular fluid below 15 GPa, gradually becoming a dissociated fluid with further compression. On the basis of modeled density and conductivity results, we suggest that partial melts containing a few percent of water may be gravitationally trapped both above and below the upper mantle-transition region. Moreover, such hydrous melts can give rise to detectable electrical conductance by means of electromagnetic sounding observations.

## Introduction

Although the majority of water in the Earth’s mantle is in minerals, water in silicate melts also plays a key role in global water cycle^1–3^. During the accretion state, large-scale magma ocean may have served as a reservoir of volatiles including water delivered to Earth. In the later stages of Earth evolution, melting is limited, mostly existing as partial melts in regions below mid-ocean ridges, regions above and below the mantle-transition region, and atop the core-mantle boundary. These hydrous silicate magmas are thus important in controlling the storage and transport of water in the interior^3^.

Water dissolved in silicate melts can significantly influence the host physical properties, for instance, by lowering the density and enhancing the electrical conductivity^4–9^. One of the major goals is to constrain the partial molar volume of water in silicate melts as a function of pressure, temperature, and composition^6,7,10,11^. This is important in evaluating the density of melts for different water contents at relevant conditions of the Earth’s interior. It is the density of silicate melts relative to the density of the surrounding mantle rocks that determines the direction and rate of melt migration and the amount of melt when partial melting occurs at a depth^12,13^. The melt-crystal density crossover may have played a crucial role in the processes that governed the fractional crystallization and chemical differentiation of early magma oceans as well as in the large fraction of geological history^3,14,15^. Like the density, the electrical conductivity of silicate melts can be compared with magnetotelluric survey data to detect and quantify possible partial melts in the mantle^16–18^.

A large number of experimental data is available for hydrous silicate melts at upper mantle-transition zone conditions as recently reviewed by Manning^19^. For instance, the measured data at pressures up to 20 GPa in the temperature range 1273–2473 K have recently been used to derive the equation of state for the partial molar volume of water (${\overline{V} }_{{\mathrm{H}}_{2}\mathrm{O}}$) in magmas^10^. While an overall decreasing trend of ${\overline{V} }_{{\mathrm{H}}_{2}\mathrm{O}}$ with pressure is evident, the extent of pressure-induced variations among different measurements and different melt compositions is large and some data even show anomalous increase with compression^10,20,21^. The measured electrical conductivity data for hydrous silicate melts are rather scarce and scattered^8,9,22,23^. One primary reason for such inconsistency and difficulty could be associated with the difficulty of quantifying the water content of the samples due to water loss during high temperature experiments.

On the other hand, a relatively few computational studies of hydrous silicate melts have so far been reported using first-principles approach^6,7,24–26^. These studies have covered relatively high temperatures and chemically simple compositions. Moreover, these computations may have been biased in a quantitative sense. For example, the use of the local density approximiation (LDA) for the exchange–correlation functional tends to overbind the structure leading to significant underestimation of the volume^6^. While the calculated partial molar volume of water appears to be mostly insensitive to water concentration^26^ and melt composition^6,24,25^, the issue is yet to be fully resolved. Similarly, it is not clear how hydrous contributions to electrical conduction are sensitive to pressure, temperature, and composition.

Here, we evaluate the partial molar volume (${\overline{V} }_{{\mathrm{H}}_{2}\mathrm{O}}$), speciation, and diffusion coefficients (and the contributions to electrical conductivity) of the water component by performing first-principles molecular dynamics (FPMD) simulations of hydrous Mg_1−*x*_Fe_*x*_SiO_3_ and Mg_2(1−*x*)_Fe_2*x*_SiO_4_ liquids for *x* = 0 and 0.25 within the generalized gradients approximation (GGA) as in our recent study^25^. GGA is generally considered to be more accurate than LDA in dealing with hydrous systems. These melt systems differ in the degree of structural polymerization with (Mg + Fe)/Si ratio of 1 and 2. We consider three different concentrations of water (6.0, 8.2, and 11.4 wt%) and wide ranges of pressure (0–140 GPa) and temperature (2000 to 4000 K). We can use the calculated properties of bulk (pure) liquid water as a basis to ascertaining the effects of water on melt properties. For instance, the molar volume of water, ${V}_{{\mathrm{H}}_{2}\mathrm{O}}$, may be taken as an upper bound on ${\overline{V} }_{{\mathrm{H}}_{2}\mathrm{O}}$. The high-pressure behavior of water is also crucial to our understanding of giant planets^27,28^ and shock wave data^29^. The pressure and temperature space considered here covers the phase transition of water from a molecular liquid to a dissociated liquid^30^. Finally, we use the simulated results to model the effects of water on the density and electrical conductivity of silicate melts and discuss their geophysical implications.

## Results and analysis

### Equation of state of hydrous silicate melts and pure water

**Figure 1:**
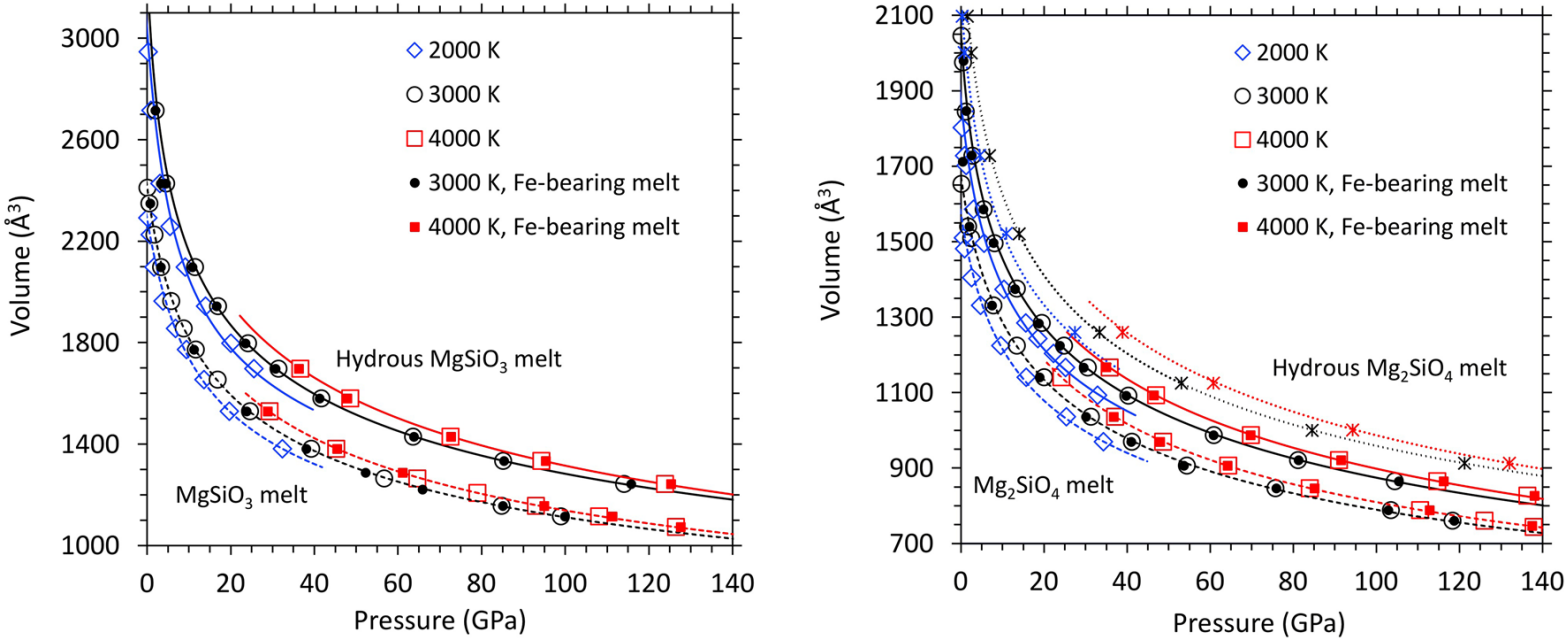
The volume-pressure profiles of hydrous MgSiO_3_ (left) and Mg_2_SiO_4_ (right) melts compared to their dry counterparts at 2000 K (blue diamonds), 3000 K (black circles) and 4000 K (red squares). Curves represent the equation of state fits using Eqs. (1) and (2). The supercells consist of 32MgSiO_3_ + 16H_2_O (8.2 wt% water) and 16Mg_2_SiO_4_ + 8H_2_O (6.0 wt% water) and corresponding iron-bearing phases at 3000 and 4000 K (small solid symbols). Also shown are the results for 16Mg_2_SiO_4_ + 16H_2_O (11.4 wt% water) at three temperatures (asterisks and dotted curves). The errors in the calculated pressure are within the size of symbols.

We present the calculated pressure–volume–temperature (*P–V–T*) results for all hydrous and dry Mg_1−*x*_Fe_*x*_SiO_3_ and Mg_2(1−*x*)_,Fe_2*x*_SiO_4_ melts for *x* = 0 and 0.25 in Fig. 1. The volume of hydrous melts is systematically larger than the volume of the corresponding water-free melts for the supercells used. Our simulations cover the pressure ranges 0 to ~ 30 GPa at 2000 K, 0 to ~ 115 GPa at 3000 K, and ~ 35 to ~ 140 GPa at 4000 K. In the overlapping pressure regimes, the volume at a higher temperature is larger than the volume at a lower temperature. The calculated results can be adequately represented using the following relation^7,13^.

1 $$P\left( {V,T} \right) = \, P\left( {V,T_{0} } \right) + \, B_{TH} \left( {V)(T - T_{0} } \right)$$

Here, the reference isotherm, *P*(*V,T*_0_) with *T*_0_ = 3000 K, is described by the 4th order Birch-Murnaghan equation with fit parameters shown in Table 1. Hydrous melts have relatively small zero-pressure bulk moduli, meaning that they are highly compressible at low pressures. The pressure–volume isotherms become nearly parallel with those of dry melts at high pressure. The thermal pressure coefficient in Eq. 1 is expressed by

2 $${B}_{\mathrm{TH}}(V)=\frac{1}{1000}\left[a+b\left(\frac{{V}_{0}}{V}\right)+c{\left(\frac{{V}_{0}}{V}\right)}^{2}+d{\left(\frac{{V}_{0}}{V}\right)}^{3}\right]$$

where *V*_0_ is the volume at zero pressure and 3000 K (Table 1). Our calculated *P–V-T* results can be also described by an alternative form for the equation of state, which is based on the hard-sphere model^31^ (Supplementary Text 1). Two forms of equation of state agree well within the interpolation pressure regime at a given temperature, however, they tend to differ considerably at extrapolated pressures (Supplementary Fig. S1).

**Table 1:** Equation of state parameters of hydrous and dry silicate melts at 3000 K. The same parameters with higher zero-pressure density as shown apply to their Fe-bearing counterparts. The parameters for melt containing 11.4 wt% water are given in the parentheses.

	Hydrous MgSiO_3_	Dry MgSiO_3_	Hydrous Mg_2_SiO_4_	Dry Mg_2_SiO_4_
${V}_{0}$(Å^3^)	3280 ± 25	2415 ± 16	2050 ± 21 (2370 ± 20)	1670 ± 14
ρ_0_ (g/cm^3^) Fe-bearing melt	1.772 1.900	2.209 2.382	1.940 (1.779) 2.144	2.238 2.489
${K}_{0}$(GPa)	8.2 ± 0.5	19.1 ± 1.0	11.3 ± 1.1 (10.1 ± 0.7)	22.2 ± 1.4
${K}_{0}{^{\prime}}$	4.85 ± 0.09	4.02 ± 0.13	5.53 ± 0.26 (5.01 ± 0.15)	3.94 ± 0.11
${K}_{0}^{{{\prime}}{{\prime}}}$(GPa^−1^)	− 0.50 ± 0.07	− 0.08 ± 0.02	− 0.69 ± 0.16 (-0.53 ± 0.10)	− 0.06 ± 0.01
*a*	26.9	32.7	19.9	7.9
*b*	− 53.8	− 70.9	− 41.6	− 23.4
*c*	34.0	49.3	28.2	21.1
*d*	− 6.1	− 9.9	− 5.2	− 4.5

While the effects of iron (with no spin state) on volume are almost negligible (Fig. 1), iron increases the melt density considerably for mass reason. Iron increases the melt density by about 7.9 and 11.2%, respectively, for Mg_0.75_Fe_0.25_SiO_3_ and Mg_1.5_Fe_0.5_SiO_4_ melts uniformly at all pressures along each isotherm (Table 1 and supplementary Fig. 2). The hydrous component, however, decreases the density much more at low pressures. The hydrous effects get rapidly diminished with increasing pressure and become gradual over much of the pressure regime (Supplementary Fig. S2). Our comparison with the measured data of silicate melts shows that GGA systematically underestimates the melt density. For instance, the calculated zero-pressure density is 2.370 g/cm^3^ compared to the experimental value of 2.583 g/cm^3^ for MgSiO_3_ melt at 1830 K and is 2.394 g/cm^3^ compared to the experimental value of 2.686 g/cm^3^ for Mg_2_SiO_4_ at 2163 K. On the other hand, LDA tends to overestimate the melt density. Nevertheless, the calculated effects of water and iron on the melt density are expected to be comparable between the two types of calculations.

**Figure 2:**
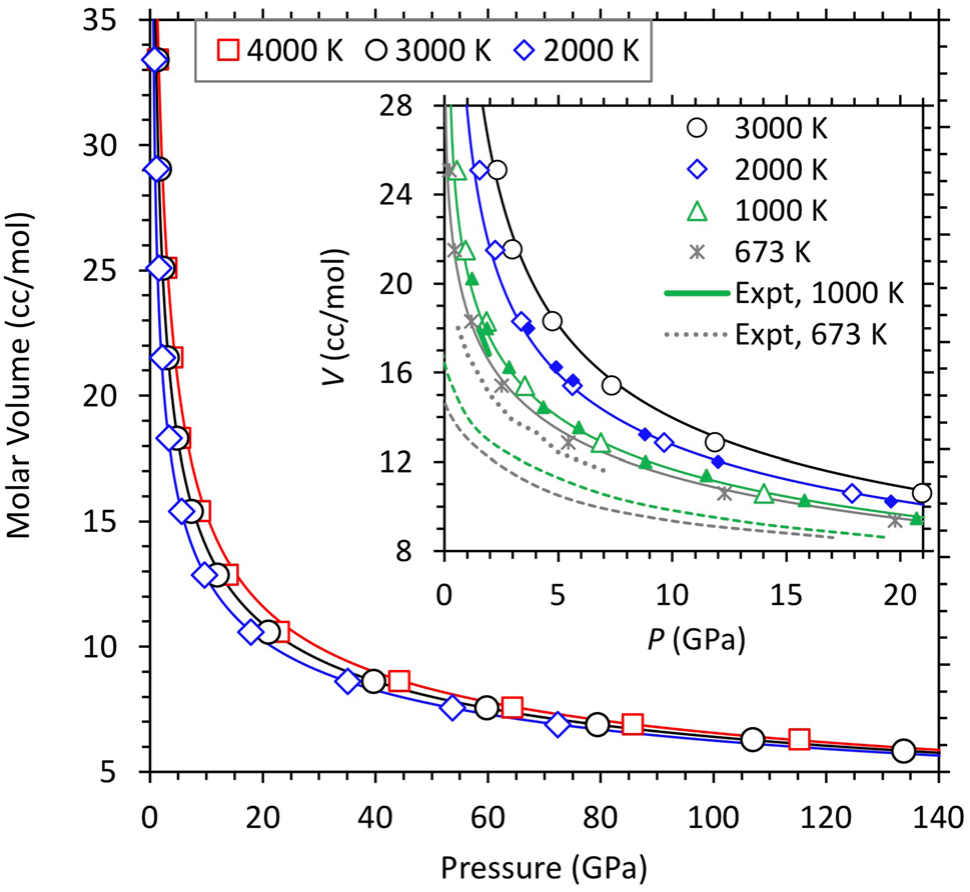
Volume-pressure relationships of pure liquid water at 2000, 3000, and 4000 K. Symbols represent the calculated values and curves represent the corresponding equation of state fits. The inset compares our calculated results (symbols and curves) with the available low-pressure experimental data at 673 and 973 K^34,35^ and the previous calculations shown by small solid triangles and diamonds^32^. Also shown are the LDA results at 673 and 1000 K (green and gray dashed lines) which are shifted downward with respect to both the GGA and experimental results. The errors in the calculated pressure are within the size of the symbols.

We represent the pressure–volume-temperature results of pure water (Fig. 2) with Eq. 1 by adding a constant pressure term (*P*_0_ = 0.5 GPa) for the reference 2000 K isotherm (Table 2). The volume-dependent thermal pressure coefficient is given by ${B}_{\text{TH}}\left(V\right)=-0.92+1.2\left(\frac{{V}_{0}}{V}\right)+0.07{\left(\frac{{V}_{0}}{V}\right)}^{2}$ in the units of mPa/K, where *V*_0_ is volume at *P*_0_. Pure water is extremely compressible as reflected by its unusually small bulk modulus. For instance, the volume along the 2000 K isotherm decreases from 39 cm^3^ mol^−1^ at 0.5 GPa to 16 cm^3^ mol^−1^ at 5 GPa. It changes more gradually as the fluid is compressed further. Different isotherms tend to become parallel and remain close to each other at high pressures. The calculated volume remains above 5.5 cm^3^ mol^−1^ at 150 GPa. Our pressure–volume results at 1000 and 2000 K are similar to the previous calculations which used GGA and relatively large supercells^32^. The experimentally inferred data on liquid water are confined to low pressures and low temperatures^33–35^. Our comparisons with the experimental data indicate that GGA overestimates the volume (lying within 5%) whereas LDA underestimates the volume (differing by up to 15%) as shown in Fig. 2. A similar degree of volume overestimation and hence density underestimation by GGA was predicted^32^ for the 300 K isotherm of ice VII/X compared to the measured data over much wider pressure range up to 126 GPa^29,36^.

**Table 2:** Equation of state parameters at 2000 K for the water dissolved in silicate melts by considering together the results for all hydrous iron-free melts (MgSiO_3_ with 8.2 wt% water, Mg_2_SiO_4_ with 6.0 and 11.4 wt% water). The experimentally inferred parameters are from Sakamaki^10^. The parameters for pure liquid water correspond to a non-zero constant pressure (0.5 GPa).

	Water in silicate melts		Pure Water
	Calc	Expt	Calc
${T}_{0}$ (K)	2000	2000 (1273)	2000
${V}_{0}$ (cm^3^/mol)	25.0 ± 0.6	35.5 (23.8)	39.0 ± 1.5
${K}_{0}$ (GPa)	1.91 ± 0.28	1.23 (1.18)	0.72 ± 0.10
${K}_{0}^{^{\prime}}$	5.80 ± 0.23	3.31 (3.35)	4.13 ± 0.05
${K}_{0}^{{{\prime}}{{\prime}}}$(GPa^−1^)	− 5.1 ± 1.5	− 2.81 (− 2.95)	− 5.2 ± 1.0

### Partial molar volume of dissolved water

**Figure 3:**
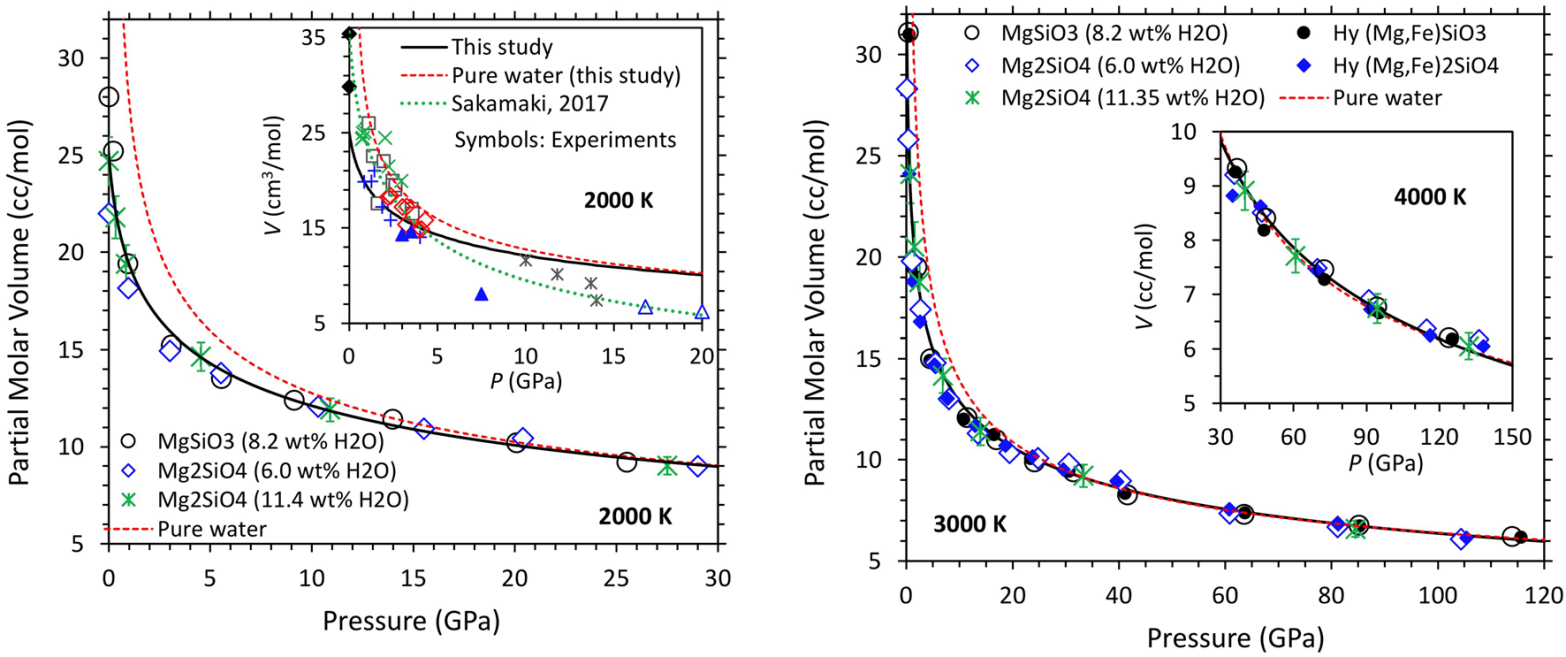
Calculated partial molar volume of water in hydrous MgSiO_3_ enstatite melt (circles) and Mg_2_SiO_4_ forsterite melts (diamonds and asterisks) as a function of pressure at 2000, 3000, and 4000 K and hydrous Mg_0.75_Fe_0.25_SiO_3_ (small solid circles) and Mg_1.5_Fe_0.5_SiO_4_ (small solid diamonds) at 3000 and 4000 K. Also shown is the equation of state fit (solid curve) to all melt results collectively at each temperature in comparison with the calculated equation of state for pure water (this study). The inset on left shows the experimental data on the partial molar volume of water in several silicate melts in the temperature range 1273–2473 K projected to 2000 K using the model of Sakamaki^10^. Different symbols represent different melt compositions, plus: andesite^39^, solid triangle: basalt^12^, square: granite^40^, open triangle: komotiite^41^, open diamond: peridotite^5^, cross: phonolite^42^, and asterisk: ultramafic^4,31^; solid diamond: hydrous silicate melts at 0 GPa^20,21^. The error bars are shown only for the hydrous forsterite results (asterisks) for the sake of clarity.

We evaluate the partial molar volume of water in silicate melt (${\overline{V} }_{{\text{H}}_{2}{\text{O}}}$) from the direct volume difference between hydrous melt (*V*_hymelt_) and dry melt (*V*_melt_):

3 $${\overline{V} }_{{\text{H}}_{2}{\text{O}}}(P,T)=\left({V}_{\text{hymelt}}(P,T)-{V}_{\mathrm{melt}}(P,T)\right){N}_{A}/n$$

where *n* is the number of H_2_O units in the supercell and *N*_A_ is the Avogadro’s number. Figure 3 shows the calculated partial molar volume of water in hydrous MgSiO_3_ and Mg_2_SiO_4_ melts and in their iron-bearing phases. We model the compression behavior of ${\overline{V} }_{{\text{H}}_{2}{\text{O}}}$ using Eq. (1). The reference isotherm *P*(*V,T*_0_) with *T*_0_ = 2000 K considering all results for three iron-free melts (Fig. 3, left) is described by the 4th order Birch-Murnaghan equation of state (Table 2). The zero-pressure bulk modulus is small, consistent with experimentally inferred value^10^. The thermal pressure coefficient in the 2000–4000 K range can be described by ${B}_{\text{TH}}\left(V\right)=0.3-0.8\left(\frac{{V}_{0}}{V}\right)+0.9{\left(\frac{{V}_{0}}{V}\right)}^{2}$ in the units of mPa/K. For each hydrous silicate melt, ${\overline{V} }_{{\text{H}}_{2}{\text{O}}}$ decreases rapidly initially with increasing pressure and then decreases more gradually at higher pressures. Hydrous enstatite melts tend to have larger ${\overline{V} }_{{\text{H}}_{2}{\text{O}}}$ than hydrous forsterite melts at pressures close to zero, but they have similar values over much of the pressure regime explored. It is also remarkable that the iron-induced differences in ${\overline{V} }_{{\text{H}}_{2}{\text{O}}}$ are negligible at all conditions (Fig. 3, right). Our results thus suggest that the partial molar volume of water remains insensitive to melt composition within the computational uncertainty over almost entire mantle pressure regime (except at low pressures up to 5 GPa).

We compare the calculated ${\overline{V} }_{{\text{H}}_{2}{\text{O}}}$ of hydrous melts at 2000 K with the experimental data in the temperature range 1273 to 2473 K at pressures up to 20 GPa in the inset of Fig. 3, left. The experimental melt compositions are more complex, including albite^37^^.^^38^, andesite^39^, basalt^12^, granite^40^, komotiite^41^, peridotite^5^, phonolite^42^, rhyolite^20^, and ultramafic^4,31^. The reported values at the ambient pressure and 1273 K for ${\overline{V} }_{{\text{H}}_{2}{\text{O}}}$ and its temperature derivative ($\partial {\overline{V} }_{{\text{H}}_{2}{\text{O}}}/\partial T$) are 22.9 cm^3^ mol^−1^ and 9.5 $\times$ 10^–3^ cm^3^ mol^−1^ K^−1^, respectively^20^. More recently reported values^21^ are 23.8 cm^3^ mol^−1^ and 15.9 $\times$ 10^–3^ cm^3^ mol^−1^ K^−1^. These studies have suggested that the partial molar volume of water is independent of the melt composition and water concentration. Using these two sets of the parameters, we estimate ${\overline{V} }_{{\text{H}}_{2}{\text{O}}}$ to be 29.8 and 35.4 cm^3^ mol^−1^ at 2000 K and 0 GPa. They are larger than our predicted values of 28.0 cm^3^ mol^−1^ for hydrous enstatite melt and 23.5 cm^3^ mol^−1^ for hydrous forsterite melt.

Comparison of the calculated partial molar volume of water in silicate melts (${\overline{V} }_{{\text{H}}_{2}{\text{O}}}$) with the molar volume of pure liquid water (${V}_{{\text{H}}_{2}{\text{O}}}$) allows us to assess the nature of mixing between the volatile and silicate components. As shown in Fig. 3, ${\overline{V} }_{{\text{H}}_{2}{\text{O}}}$ is smaller than ${V}_{{\text{H}}_{2}{\text{O}}}$ at zero pressure but the two volumes approach each other rapidly with increasing pressure. The apparent volume of the melt + water solution is large negative (− 18.8 cm^3^ mol^−1^ at 0.5 GPa and 2000 K) and remains negative at pressures below 15 GPa, but it becomes almost zero on further compression. The volume of mixing thus changes from non-ideal to ideal with increasing pressure. Non-ideal mixing of silicate melts and H_2_O fluid was also implied experimentally at low pressures^43–45^.

### Hydrogen and oxygen diffusion

**Figure 4:**
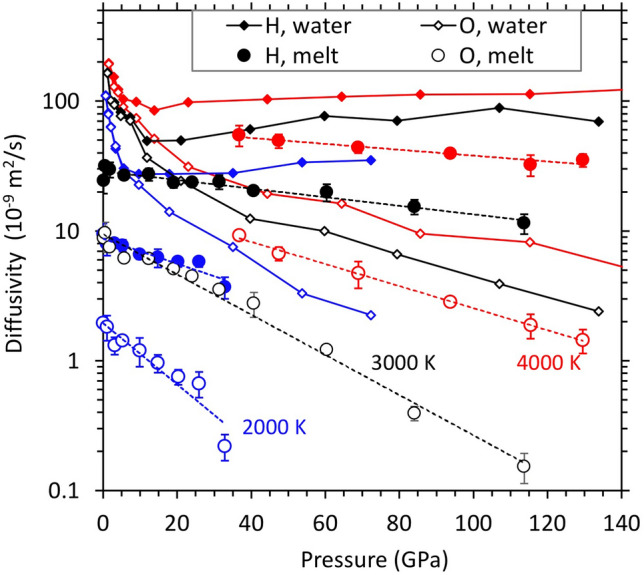
Diffusivity-pressure profiles of hydrogen and oxygen of hydrous silicate melts (symbols) taken as averages over five melt compositions (with the corresponding error bars shown) at three temperatures. Their Arrhenius trends are represented by straight dashed lines. The H and O diffusivity results of pure water are displayed, respectively, as solid and dashed curves (with small symbols). Color encodes temperature: blue 2000 K, black 3000 K, and red 4000 K.

We find that hydrogen is highly mobile in all melts simulated here, in general agreement with previous computational results^26,46,47^. At each temperature, the diffusivity decreases with increasing pressure implying positive activation volume and all log*D*_H_–*P* isotherms tend to be linear (Fig. 4). Comparison of these isotherms among different melts suggests that hydrogen diffuses somewhat faster in hydrous silicate melts with higher water content (Supplementary Fig. S3). The presence of iron tends to enhance hydrogen diffusion and diminish the differences in *D*_H_ between the two silicate melt systems (Supplementary Fig. S3). Oxygen diffuses much slower than hydrogen as expected (Fig. 4). At 2000 K, the *D*_H_/*D*_O_ ratio increases from ~ 4 to more than 10 over the pressure range 0–30 GPa. At 3000 K, this ratio exceeds 10 above 50 GPa and becomes so large at 80 GPa that O atoms (as well as Mg and Si atoms) can be essentially assumed as frozen with respect to H. At 4000 K, all atomic species including oxygen diffuse at discernable rates even at very high pressures. Our results thus suggest that hydrogen diffuses a few orders of magnitude faster than the host ions in silicate melts even at the conditions applicable to the deepest parts of the mantle.

In pure liquid water, hydrogen and oxygen diffuse in a different manner than in silicate melts. As pressure increases, both diffusivities (*D*_H_ and *D*_O_) decrease rapidly almost overlapping with each other in low-pressure regime (Fig. 4), that is, the *D*_H_/*D*_O_ ratio remains close to 1 implying the diffusion of individual H_2_O molecules at pressures up to 10 GPa. The simulated water is thus considered to be a molecular fluid at low pressures irrespective of temperature. As pressure increases further, hydrogen and oxygen diffusion decouple from each other. The *D*_H_ profile reaches a shallow minimum before it shows a gradual increasing trend at high pressures. On the other hand, the oxygen diffusivity continues to decrease with increasing pressure. The two diffusivity profiles systematically diverge with compression, for instance, the *D*_H_/*D*_O_ ratio is ~ 16 at 72 GPa (2000 K) and ~ 24 at 142 GPa (4000 K). Because hydrogen diffuses much faster than oxygen, the simulated water essentially represents a dissociated fluid state over much of the pressure regime studied.

We find that the calculated hydrogen and oxygen diffusivities of hydrous silicate melts are much smaller than those of the bulk water particularly at low temperatures (Fig. 4). Also, melt *D*_H_ and *D*_O_ differ from each other at all pressures, including zero pressure. Melt *D*_H_ decreases gradually with pressure with no anomalous behavior shown by water *D*_H_. Melt *D*_O_ decreases monotonically with pressure though more rapidly than water *D*_O._ In silicate melt, oxygen diffusion is also controlled by Mg–O and Si–O bond events. Our results show that the diffusivity isotherms of hydrous silicate melts and pure water differ more as pressure increases.

### Speciation of hydrous component and structure of water

**Figure 5:**
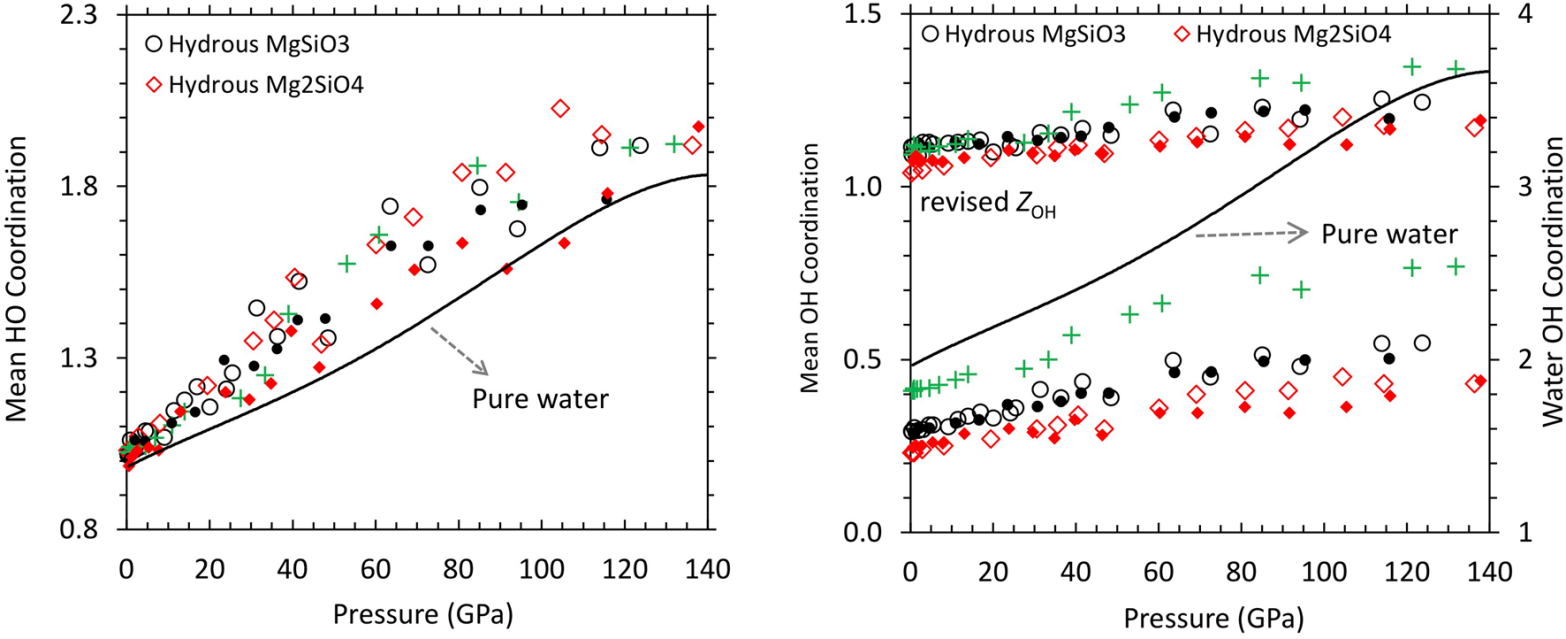
Mean H–O and O–H coordination numbers of hydrous MgSiO_3_ with 8.2 wt% water (circles) and Mg_2_SiO_4_ with 6.0 wt% water (diamonds) and 11.4 wt% water (pluses) compared to those of pure water (solid curve). The revised *Z*_OH_ (right) represents the mean coordination number evaluated by considering only oxygen atoms that contribute to the coordination. The results for the corresponding iron-bearing phases are shown by small solid symbols.

Hydrogen exclusively forms bond with oxygen in hydrous silicate melts as in bulk water. This is reflected by a sharp peak around 1 Å in the H–O radial distribution function and a broad peak in all other hydrogen-involved functions (Supplementary Fig. S4 and S5). So, the speciation of hydrous component in melts is determined by the local coordination environments of H and O. The calculated mean H–O coordination number (*Z*_HO_) of hydrous silicate melts is slightly larger than 1 at zero pressure and its value increases almost linearly with pressure along each isotherm (Fig. 5, left). Melt *Z*_HO_ always remains above water *Z*_HO_ (which is 1 at zero pressure) with larger differences between them appearing at higher pressures. At zero pressure, we find that a small proportion (2–3%) of hydrogen atoms are coordinated with two oxygen atoms (that is, forming –O–H–O– bridge) in melts whereas all hydrogen atoms are singly oxygen-coordinated in water. The proportion of twofold and onefold coordination states increases and decreases, respectively, with increasing pressure more rapidly in melts compared to water (Supplementary Fig. S6). This difference arises partly because more oxygen atoms are available in hydrous melts than in pure water for hydrogen to form H–O bond. In the case of iron-bearing silicate melts, hydrogen has an affinity for iron, which is manifested by a shoulder-like feature in the H–Fe radial distribution function (Supplementary Fig. S4). The associated H–Fe bonding tends to cause a slight reduction in *Z*_HO_.

**Figure 6:**
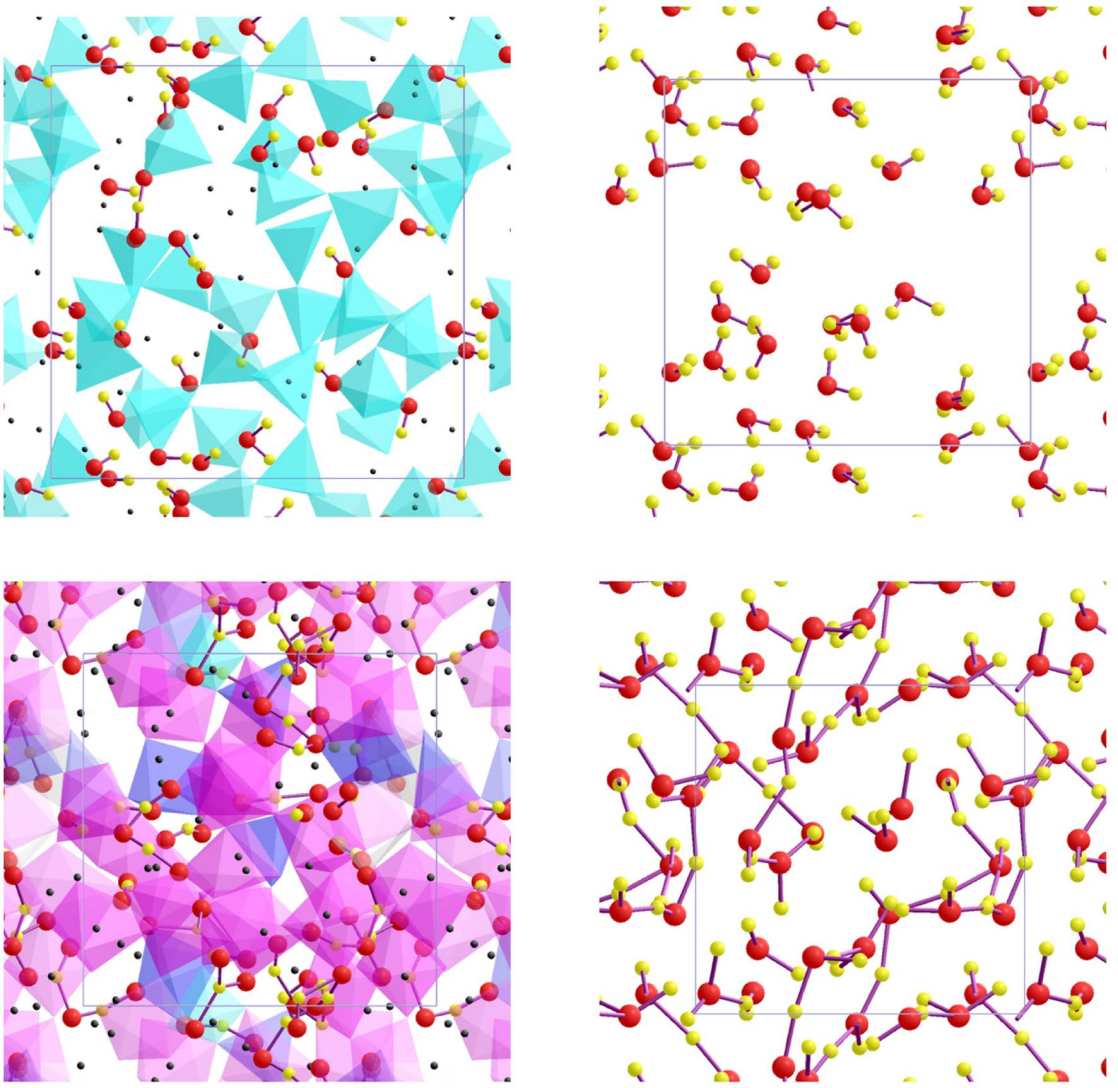
Visualization snapshots of hydrous silicate melt (32 MgSiO_3_ + 16 H_2_O) and bulk water (24 H_2_O) at low and high pressures^60﻿^. The hydrous melt consists of mostly hydroxyls with one H_2_O molecule and one –O–H–O– bridge at 0.2 GPa and 2000 K (top-left). It shows fewer hydroxyls, many –O–H–O– bridges, and some longer sequences (–O–H–O–H–O–, –O–H–O–H–O–H–O–, HO_3_) at 85.1 GPa and 3000 K (bottom-left). Also shown are the Si–O coordination states (cyan: tetrahedron, blue: pentahedron, magenta: octahedron) and Mg atoms (black dots). The simulated pure water contains only water molecules at 0.7 GPa and 2000 K (top-right). A highly compressed water (79.4 GPa and 3000 K) contains ten OH_2_, twelve OH_3_, and two OH_4_, which are also interconnected with each other via hydrogen (bottom-right).

On the other hand, the O–H coordination environment is sensitive to the Mg/Si ratio of melts. The calculated mean O–H coordination number (*Z*_OH_) at zero pressure is 0.29 for hydrous MgSiO_3_ melt and 0.23 for hydrous Mg_2_SiO_4_ melt. Its value increases with increasing pressure for both melts, and the differences between two melts persist at all pressures (Fig. 5, right). As expected, a large fraction of oxygen (~ 80%) in melts is not bonded with hydrogen. The rest of oxygen form mostly hydroxyls and some molecular water at low pressure (Fig. 6), generally consistent with the experimental observations^48,49^. In contrast, every oxygen is bonded with two hydrogen atoms in water corresponding to *Z*_OH_ = 2 (and *Z*_HO_ = 1) and nearly 100% H_2_O molecules at zero pressure (Fig. 6). On compression, oxygen is increasingly bonded with hydrogen in water and *Z*_OH_ exceeds 3 at high pressures. The proportion of H_2_O molecules in water decreases rapidly as pressure increases (Supplementary Fig. S6), dropping to ~ 85% at 20 GPa, then to ~ 50% at 50 GPa and ~ 10% above 100 GPa. Larger groups, such as OH_3_, OH_4_ and OH_5_ all become increasingly abundant at higher pressure (Supplementary Fig. S6). Moreover, they are linked with each other to form more complex species as hydrogen is shared with two or even three oxygens because of space confinement at high pressures. A clear structural change thus occurs from a molecular liquid to a dissociated liquid (with larger OH_n_ groups and probably more ionic in nature) as the liquid water is compressed (Fig. 6). Two oxygen coordinated states of hydrogen (that is, –O–H–O–) facilitate hydrogen movement in both water and hydrous silicate melts. These hydrogen bridges act as transition state for hydrogen transfer from one hydroxyl to another hydroxyl via the reaction O–H + O = O–H–O = O + H–O, where oxygen is also bonded with the other cation (Si or Mg in hydrous melts or hydrogen in the pure water).

Pressure systematically enhances the degree of oxygen–hydrogen bonding in hydrous silicate melts. Considering only oxygen atoms which are bonded with hydrogen, the revised value of *Z*_OH_ increases from ~ 1.1 to ~ 1.25 for hydrous MgSiO_3_ melt and from ~ 1.05 to ~ 1.20 for hydrous Mg_2_SiO_4_ melt over the pressure ranges considered here (Fig. 5, right). The majority of oxygen (80–95%) are singly coordinated with hydrogen in silicate melts. The proportion of singly and doubly hydrogen-coordinated species (that is, hydroxyls and OH_2_) gradually decreases and increases, respectively, with increasing pressure (Supplementary Fig. S6). Even hydronium group (OH_3_) appears in discernable amounts (up to ~ 3%) above 30 GPa. This pressure enhancement of twofold coordination states comprising of hydrogen and oxygen (that is, –O–H–O– and –H–O–H– groups) in silicate melts indicates a changing role of hydrogen from a network modifier at low pressure to a network former at high pressure (Fig. 6). The predicted pressure evolution of the hydrous speciation is generally consistent with previous computational studies of hydrous silicate melts^6,7,24–26^.

## Discussion and implications

### Melt + water solution

The predicted negative volume of silicate melt + water solution at low pressures (for instance, − 19, − 2.7, and − 0.7 cm^3^ mol^−1^, respectively, at 0.5, 5, and 10 GPa at 2000 K) can be attributed to the differences in local hydrogen–oxygen arrangement between silicate melt and pure water. The water component is dissolved in silicate melts primarily as hydroxyls and to some extent as molecular water, which are also bound to one or more cations via oxygen bonding (Fig. 6, left)). This means that the incorporation mechanism is predominantly chemical. On the other hand, the bulk water is in molecular liquid state exclusively consisting of loosely (physically) bound H_2_O molecules at low pressures (Fig. 6, top-right)). Such a molecular fluid water is highly compressible, so its molar volume becomes comparable to the partial molar volume of the dissolved water of the melt at pressures above 15 GPa (Fig. 3). The pressure-induced similarity between two volumes can be associated with our finding that the H_2_O component in melts as well as the bulk water become structurally well-connected (that is, exclusively chemical) at high compression. Enhanced participation of oxygen in hydrogen bonding becomes notable and also the proportions of species, such as H_3_O and H_4_O, are large in highly compressed bulk water, which represents the dissociation of water as in the case of hydrous melts. The system thus shows increasingly ideal volume of mixing with compression. This explains the experimental observations suggesting that water and silicate melt become completely miscible at high pressures^45^. Our finding that hydrous melts of different compositions (varying Mg/Si and iron content) behave similarly at all pressures (Fig. 3) suggests that the predicted effects of hydration on melt properties are likely to be applicable to natural melt compositions as well.

Most previous first-principles computations used LDA to study silicate melts, including hydrous ones^6,7,46,47^. Comparison of our new results with the previous results allow us to assess how much the evaluation of partial molar volume is sensitive to the choice of the exchange correlation functional (Supplementary Fig. S7). A general trend is that GGA gives systematically larger volume than LDA. This is indeed the case with both pure and hydrous silicate melts. The volume difference between GGA and LDA for these melts is large at zero pressure (~ 15%) and it decreases gradually with increasing pressure (~ 5% at 50 GPa). This trend is also evident in the bulk water for which the volume difference is very large particularly at low pressures (~ 50% at 0.5 GPa, ~ 20% at 5 GPa), dropping below 5% at pressures beyond 100 GPa. In the case of the partial molar volume of water, the GGA-LDA difference is large at low pressures but it almost vanishes at high pressures (Supplementary Fig. S7). As discussed earlier, our GGA prediction is expected to be more accurate than the previous LDA results in case of the molar volume of the dissolved water.

### Density change by hydration

**Figure 7:**
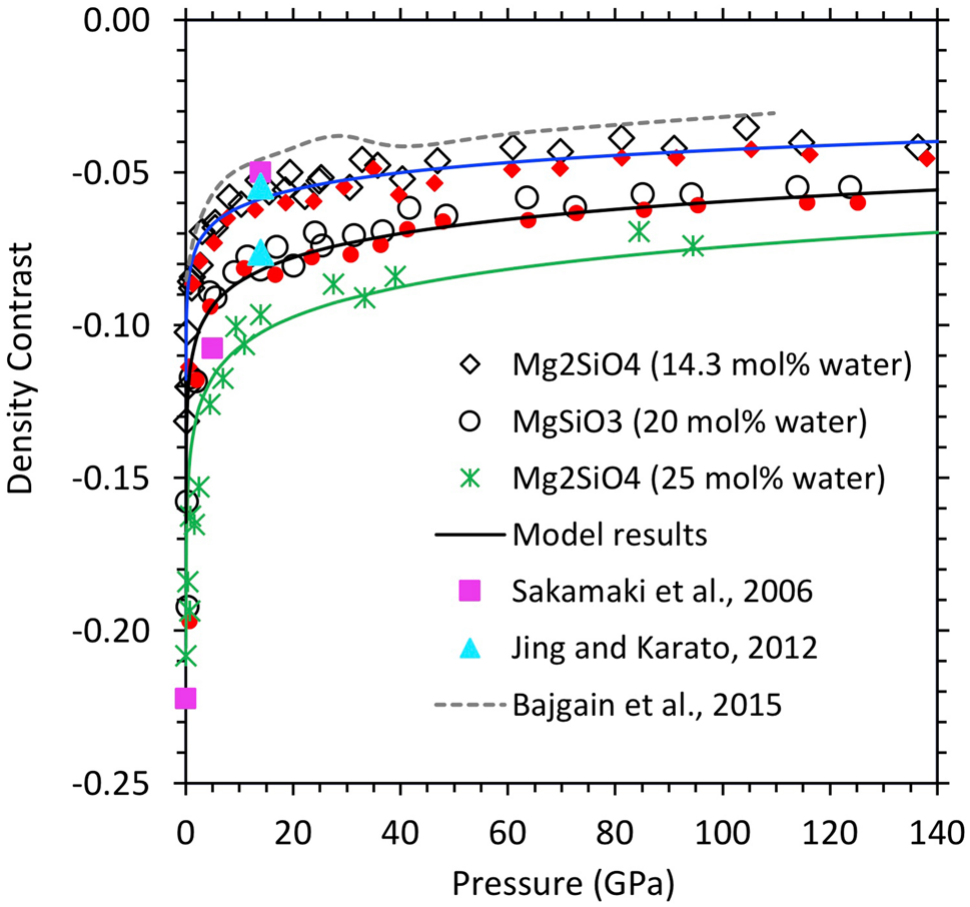
Density contrast between dry and hydrous melts for iron-free (open symbols and asterisks) and iron-bearing (filled small symbols) compositions as a function of pressure. The model results using Eq. 4 are shown by solid curves for the corresponding melt compositions. The previous results are shown for peridotite melt^12^ containing ~ 20 mol% water at 0, 5, and 14 GPa at 1873 K (magenta squares), hydrous ultramafic melt^31^ with 20 and 25 mol% water at 14 GPa and 2173 K (cyan triangles), and hydrous model basalt^6^ with 13 mol% water along the 3000 K isotherm (dashed curve).

The water-induced reduction in the melt density tends to be nearly uniform with respect to pressure and temperature^6,7,24^. The calculated density contrasts (Fig. 7) can be adequately described with the following relation:

4 $$\frac{\rho -{\rho }_{0}}{{\rho }_{0}}=\left[(-0.56\pm 0.01)+(0.057\pm 0.001)\mathrm{ln}\left(P\right)\right]{x}_{\text{m}}/100$$

where *ρ* is the density of hydrous silicate melt, ${\rho }_{0}$ is the density of non-volatile component (dry silicate melt), and ${x}_{\mathrm{m}}$ is the mol percent of water in terms of oxide components. Our results are generally comparable with the previous estimates of the density contrasts based on the experimental data^6,12^ and first-principles simulations^6^ for more complex melt compositions (Fig. 7). These studies implied somewhat smaller density effects because they also inferred smaller partial molar volume of water dissolved in melts as discussed earlier (Fig. 2, left-inset). We find that the reduction in melt density by hydration depends linearly on water concentration. The density contrast varies gradually with pressure according to Eq. (4) (over much of the pressure regime except below 5 GPa), which is independent of temperature and melt composition within the computational uncertainty (Fig. 7, Supplementary Fig S8). Our model differs from the previous model^24^ which assumed that the density contrast only depends on water content in terms of weight percent. We find that the amount of water in terms of mol percent determines the magnitude of density contrast more accurately than that in terms of weight percent. This perhaps makes sense because the density changes are also related to the dilation caused by the hydrous component. If the melt + water solution has zero volume of mixing at all conditions, the density contrast between hydrous and anhydrous melts will vary rapidly initially and then gradually with pressure^7^. The predicted hydration density at low pressures is smaller than that in an ideal mixing case.

Silicate melts were prevalent in magma ocean environment soon after Earth was formed. Now they are present to much smaller extents, most likely in regions below mid-ocean ridges, regions above and below the mantle-transition region, and atop the core-mantle boundary. A melt formed just above the mantle transition zone can be gravitationally stable if the melt density takes a value between the density of the upper mantle minerals near 410 km depth (3.54 g/cm^3^) and the density of the materials at the top of mantle transition zone (3.73 g/cm^3^). Using Eq. 4 with a density contrast evaluated^50^ as (3.54–3.73)/3.73 = − 0.051, we obtain *x*_m_ = 12.4 mol% at 14 GPa. This means that a melt containing up to 5 wt% water will be stable atop the mantle transition zone if the FeO content of melt is high (say, Fe/(Mg + Fe) = 0.25). Similarly, we estimate that up to 9 wt% water may be accommodated in a buoyantly neutral hydrous melt at the 660 km depth discontinuity corresponding to its large density jump^50^ of 0.38 g/cm^3^. The implied presence of gravitationally trapped hydrous partial melts at these depths have been previously suggested^6,12,26,51^. The water content of possible melts at these depths also depends on the iron content because water and iron change the density in an opposite way. Both iron and water are preferably partitioned into melts when partial melting occurs at a depth or when a magma ocean starts to crystallize resulting in a dense hydrous melt.

**Figure 8:**
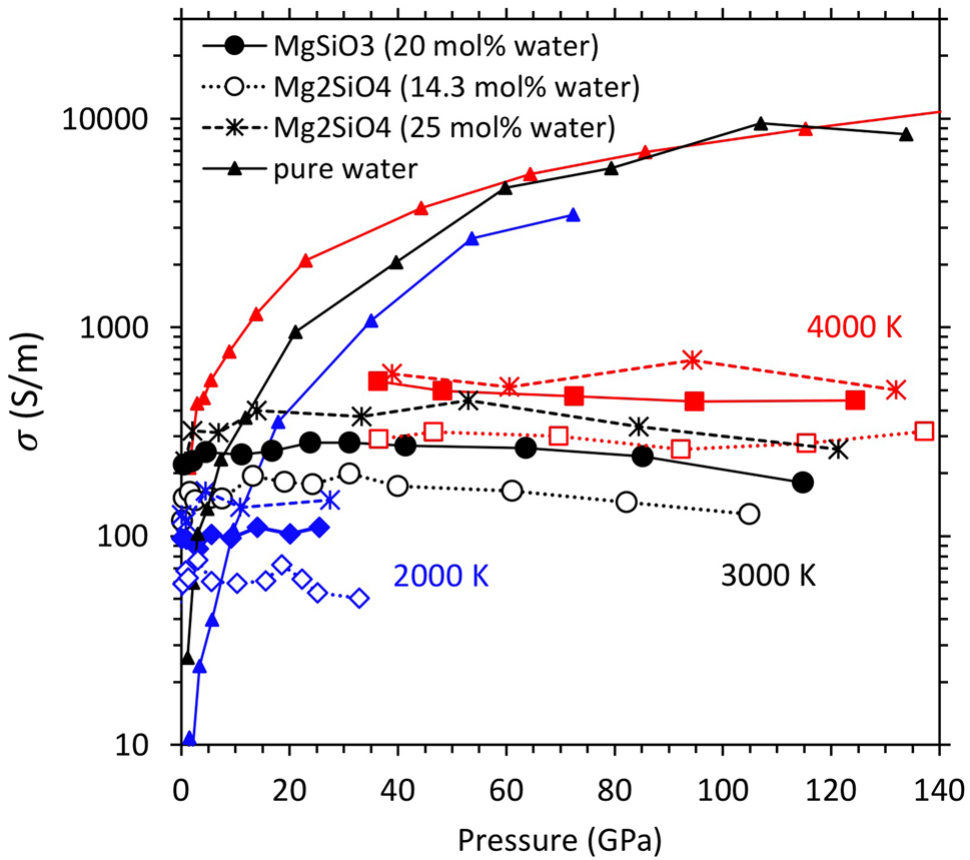
Hydrogen-induced electrical conductivity ($\sigma$) of hydrous MgSiO_3_ (filled symbols) and Mg_2_SiO_4_ melts (open symbols and asterisks) and bulk water (solid curves) as a function of pressure at different temperatures as shown. The melt conductivity at 3000 and 4000 K represent the averages taken between the iron-free and iron-bearing compositions (iron has almost no influence on the conductivity, Supplementary Fig. S9). The errors in the calculated electrical conductivity lie within 20%.

*Electrical conductivity.* Highly mobile hydrogen ions carrying positive charges (as protons) can contribute to the electrical conductivity ($\sigma$) of melts. We evaluate this contribution from the calculated diffusivity (*D*) values (Fig. 4) using the Nernst-Einstein relation as previously done^7^

5 $$\sigma =\frac{Dn{q}^{2}}{kT{H}_{R}}$$

where *n* is the number density of mobile carriers with electrical charge *q*, and *k* denotes the Boltzmann constant. The Haven ratio *H*_R_ is a correlation factor for the ionic motion and approaches one for small *n,* so we take *H*_R_ = 1. For hydrous silicate melts, we consider protons (H^+^ ions) as the primary charge carriers; *q* = + 1e. Our results show that the hydrogen-induced (ionic) conductivity of hydrous silicate melts (${\sigma }_{\text{melt}}$) takes larger value at higher temperature, but along each isotherm it remains almost constant with respect to pressure (Fig. 8; Supplementary Fig. S9). The average conductivity over the pressure ranges considered here corresponds to 100, 247, and 480 S/m, respectively, at 2000, 3000, and 4000 K for (Mg,Fe)SiO_3_ melts with 20.0 mol% water. The corresponding ${\sigma }_{\text{melt}}$ values of hydrous (Mg,Fe)_2_SiO_4_ melts are 63, 160, and 293 S/m for 14.3 mol% water and 138, 328, and 597 S/m for 25.0 mol% water. As expected, hydrous melt becomes more conductive for higher water concentration. These average conductivity values can be described by the following concentration-dependent Arrhenius model:

6 $$\sigma \left({x}_{\text{m}},T\right)={\sigma }_{0}({x}_{\text{m}})\mathrm{ exp}[-E/(RT)]$$

where ${\sigma }_{0}\left({x}_{\text{m}}\right)= -435+12150 {x}_{\text{m}}$ is the pre-exponential factor, varying linearly with the mol percent of water (*x*_m_), and *E* = 50 ± 2 kJ/mol is the activation energy which is independent of water content. The model predicts a value of 43 S/m at 1873 K and 14 GPa, which compares well with the experimentally inferred value^8^ of 37 S/m.

In pure water, hydrogen diffuses primarily as neutral H_2_O molecules at low pressure. We exclude the hydrogen atoms of molecular water by approximating the effective concentration of charge carriers by (1-*f*)*n*, where *f* is the proportion of H_2_O species. The estimated electrical conductivity of bulk water (${\sigma }_{\text{water}}$) increases rapidly initially and then gradually as pressure increases (Fig. 8). Our results compare favorably with previous computational results^30,52^ and measured shock wave data^53,54^. The predicted pressure behavior represents ionic conduction as water upon compression changes from an insulating molecular liquid to a fully dissociated ionic liquid. Highly compressed water behaves as a superionic liquid in which oxygen ions essentially become frozen compared to highly mobile hydrogen ions (*D*_O_ is one to two orders of magnitude smaller than *D*_H_ as shown in Fig. 4). We find that ${\sigma }_{\text{water}}$ is smaller than ${\sigma }_{\text{melt}}$ at pressures up to 10 GPa when pure water behaves as a molecular fluid (Fig. 8). As the water is compressed further, ${\sigma }_{\text{water}}$ exceeds ${\sigma }_{\text{melt}}$ because hydrogen ion diffuses via O–H bond breaking/formation events. Both the concentration and diffusion rate of charge carriers (H^+^) in compressed bulk water are high compared to those in hydrous silicate melts.

We estimate the electrical conductivity of a gravitationally stable partial melt layer at 410 km depth containing 5 wt% water as discussed above. This corresponds to *x*_m_ ~ 13 mol% for which using Eq. 6 gives ${\sigma }_{\text{melt}}$= 39 S/m at 1800 K. So, a partial melt layer containing a few percent (say, 3 volume %) of hydrous melt corresponds to a high conductivity of ~ 0.8 S/m using the Hashin–Shtrikman scheme with the assumption that the electrical conductivity of solid grains is negligible (Supplementary text 2). Electrical conductivities in the range 0.1 to 2.0 S/m have been reported in the upper-mantle-transition regions by electromagnetic sounding observations^16–18^.

### Summary

In most cases, silicate melts contain some water. Whether such hydrous melt sinks or rises or stagnates at a depth controls the amount of water that may be entertained in the interior and its subsequent contributions to global water cycle at all times of Earth’s history. For instance, upwelling hydrous magmas may have been contributing to the origin and maintenance of the atmosphere and hydrosphere. To better understand these issues requires a full knowledge about the behavior and properties of hydrous silicate melts at relevant pressure and temperature conditions. This study represents a major progress in this endeavor as it reports the results of first-principles molecular dynamics simulations of hydrous silicate melts covering wide ranges of pressure (up to 140 GPa), temperature (2000 to 4000 K), and composition (water content, Mg/Si ratio, iron content). Using the simulation results, we have made quantitative evaluation of the effects of water on the density and electrical conductivity of silicate melts. These effects are further linked to the thermodynamic and structural differences between water in silicate melts and water in its bulk (pure) form. The water component is less compressible than bulk water, particularly at low pressures. Water is dissolved in melts in dissociated form mostly as hydroxyls at low pressure and as extended species on further compression. Bulk water behaves differently as it changes from a fully molecular fluid to a dissociated fluid with compression. As a consequence, the volume of silicate melt + water solution is non-ideal at low pressure and becomes zero above 15 GPa, irrespective of melt composition and water concentration. Also, hydrogen diffusion is decoupled from the rest of ions including oxygen in silicate melts and the associated highly mobile protons make substantial contributions to electrical conduction. Finally, we have demonstrated that the water component controls the stability and conductance of partial melts in the mantle.

## Methods

First-principles molecuar dynamics simulations of several silicate melt + water systems were performed using the generalized gradients approximation (PBE exchange–correlation functional)^55^ and project augmented wave method^56^ as implemented in VASP^57^. All simulations were based on canonical *NVT* ensembles where the number of atoms *N*, volume *V*, and temperature *T* are fixed. The supercells consisted of 32MgSiO_3_ + 16H_2_O (*N* = 208), 16Mg_2_SiO_4_ + 8H_2_O (*N* = 136), and 16Mg_2_SiO_4_ + 16H_2_O (*N* = 160) for hydrous silicate melts containing 8.2, 6.0, and 11.4 wt% water (equivalently, 20.0, 14.3 and 25.0 mol% water), respectively. The corresponding water-free supercells involved 32MgSiO_3_ and 16Mg_2_SiO_4_. To simulate iron-bearing melts, Mg atoms were substituted with Fe atoms for *x* = Fe/(Mg + Fe) = 0.25. A plane wave cutoff of 400 eV and Gamma Point Brillouin zone sampling were used. Pulay stress added to the calculated pressure varies between ~ 3 and ~ 8 GPa over the volume range *V*/*V*_0_ = ~ 1.0 to ~ 0.4 (where *V*_0_ is the zero-pressure volume shown in Table 1) considered in this study.

The input configurations for each melt composition at different volumes were generated at temperatures well above the melting point followed by quenching to desired lower temperatures of 4000, 3000, and 2000 K. The run duration varies between 20 and 80 picoseconds with a time step of 1 femtosecond depending on volume and temperature. The liquid state was confirmed in each case by assuring that the radial distribution functions show no long-range order and the mean square displacement functions show diffusive regime^58^. We also performed a few simulations using the local density approximation^59^. The calculated results were assured to be adequately converged with respect to run duration, time step, and supercell size by performing additional simulations at selected conditions. For comparisons, pure fluid water was simulated using 24 formula units (*N* = 72), a plane wave cutoff of 600 eV, and a timesetp of 0.5 femtosecond. The applied Pulay stress varies from 0.2 to 0.8 GPa over the sixfold compresson regime considered. Further details about the simulation and data analysis methods can be found in previous publications^6,25,58^

## Supplementary Information

Supplementary Information.
