# Supplementary material for: Behavior and properties of water in silicate melts under deep mantle conditions
Source: Sci Rep. 2021 May 19;11:10588. doi: 10.1038/s41598-021-90124-7 (PMC8134574; doi:10.1038/s41598-021-90124-7)
Supplement: Supplementary file 1 — Supplementary Information. [file 41598_2021_90124_MOESM1_ESM.pdf]

Supplementary Information

# Behavior and properties of water in silicate melts under deep mantle conditions

Bijaya B. Karki, Dipta B. Ghosh, Shun-ichiro Karato

**Supplementary text 1.** Hard sphere-based equation of state

In the hard sphere (HS) model[1,2], the liquid is considered as a mixture of deformable spheres corresponding to different oxide components of silicate melts which are MgO, FeO, $SiO_2$ and $H_2O$. The equation of state is expressed as

$$P = \frac{RT}{V}\left[(1-\xi)\Phi - \Phi_0\left(\frac{V_0}{V}\right)^{1/3} + \xi\Phi_0\left(\frac{V_{m0}}{V_m}\right)^{5/3}\right]$$

Here $\Phi$ represents the excluded volume effect and is expressed as

$$\Phi = \frac{1+f+f^2}{(1-f)^3}$$

with packing fraction $f = V_m/V$ relating the molar volume of liquid ($V$) to the volume occupied by a mole of spheres ($V_m = \pi\sigma^3 N_A/6$). Here we adopt average sphere diameter $\sigma$ and deformability factor $\xi$.

The sphere diameter is considered to be temperature and volume dependent[1]:

$$\sigma = \sigma_0\left(\frac{T}{T_0}\right)^{\eta} \exp[\alpha_0\xi(T-T_0)/3]\left(\frac{V}{V_0}\right)^{\xi/3}$$

Here $\alpha_0$ is the coefficient of thermal expansion at zero pressure. The factor $\xi$ represents the deformability of spheres. Its value is 0 for hard sphere liquid. If $\xi$ = 1, liquid behaves as a solid because spheres and liquid are equally compressible. Our fitting shows that the model requires smaller and more deformable spheres for hydrous melts compared to dry melts because $H_2O$ corresponds to the smallest sphere among all oxide components. For each melt, the sphere diameter tends to decrease somewhat with temperature, but it decreases considerably with pressure because of non-zero values of $\xi$ in the range 0.42 and 0.54. The hard-sphere equation of parameters are given in table below.

| | $32MgSiO_3$ | $32MgSiO_3$+$16H_2O$ | $16Mg_2SiO_4$ | $16Mg_2SiO_4$+$8H_2O$ |
|---|---|---|---|---|
| $V_0$ (Å$^3$) | 2415 | 3280 | 1670 | 2050 |
| $\sigma$ (Å) | 3.152 | 2.396 | 3.104 | 2.410 |
| $\eta$ | -0.05 | -0.05 | -0.05 | -0.05 |
| $\xi$ | 0.52 | 0.42 | 0.54 | 0.49 |
| $\alpha_0$ ($\times 10^{-5}$ K$^{-1}$) | 5.6 | 8.8 | 7.8 | 10.0 |

**Supplementary text 2.** Estimation of the electrical conductivity of a partial melt layer in deep mantle.

The effective electrical conductivity of a partial melt layer ($\sigma_{\mathrm{bulk}}$) can be evaluated by the Hashin-Shtrikman upper bound[3] which assumes that spherical grains with negligible (zero) electrical conductivity are surrounded by conductive melt:

$$\sigma_{\mathrm{bulk}} = \left(\frac{2\phi}{3-\phi}\right)\sigma_{\mathrm{melt}}$$

where $\sigma_{\mathrm{melt}}$ and $\phi$ represent the electrical conductivity and volume fraction of melt, respectively.

Alternatively, we can use the cubic model[4] which assumes that cubic (non-conductive) grains are surrounded by conductive melt:

$$\sigma_{\mathrm{bulk}} = \left[1-(1-\phi)^{2/3}\right]\sigma_{\mathrm{melt}}$$

For $\sigma_{\mathrm{melt}}$ = 39 S/m and $\phi = 0.03$ corresponding to the 410 km depth and 1800 K, these models predict a bulk electrical conductivity of ~0.79 and 0.78 S/m, respectively.

**Supplementary figure S1.** Comparison between the hard-sphere (HS) and Birch-Murnaghan (BM) equation of state fits to the calculated pressure-volume results of hydrous enstatite melt (left) and hydrous forsterite melt (right) at 2000, 3000 and 4000 K. The open circles represent the calculated results at 3000 K and small filled circles represent the calculated results at 2000 and 4000 K. Two equations of states have different predictions when the results are extrapolated to the pressure-temperature range outside of the conditions where calculations were made. In the Birch-Murnaghan equation of state, we truncate the free energy to finite terms in the Taylor expansion with respect to volumetric strain. In the hard-sphere model, there is no truncation issue, but there are some assumptions regarding the nature of "hard sphere" as discussed earlier[1,2].

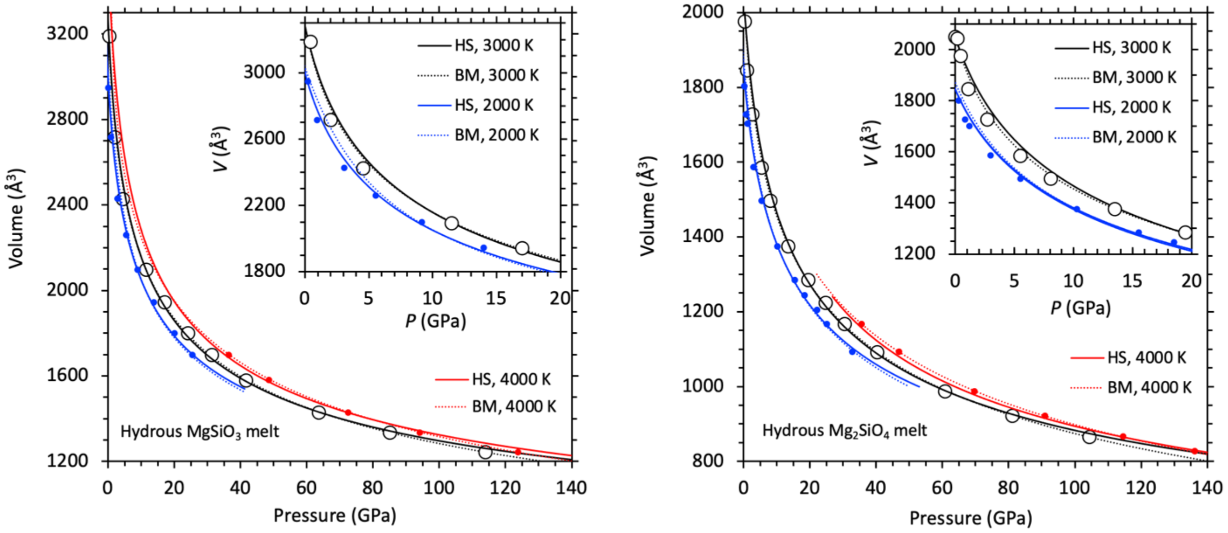

**Supplementary figure S2.** Calculated density-pressure results (symbols) of hydrous iron-free and iron-bearing silicate melts compared with those of water-free melts. The amount of iron is given by $x$ = Fe/(Mg+Fe) = 0.25. The hydrous $MgSiO_3$ and $Mg_2SiO_4$ melts (and their iron-bearing counterparts) contain 8.2 and 6.0 wt% water, respectively. The curves represent the equation of state profiles based on the parameters given in Table 1 for iron-free and iron-bearing cases.

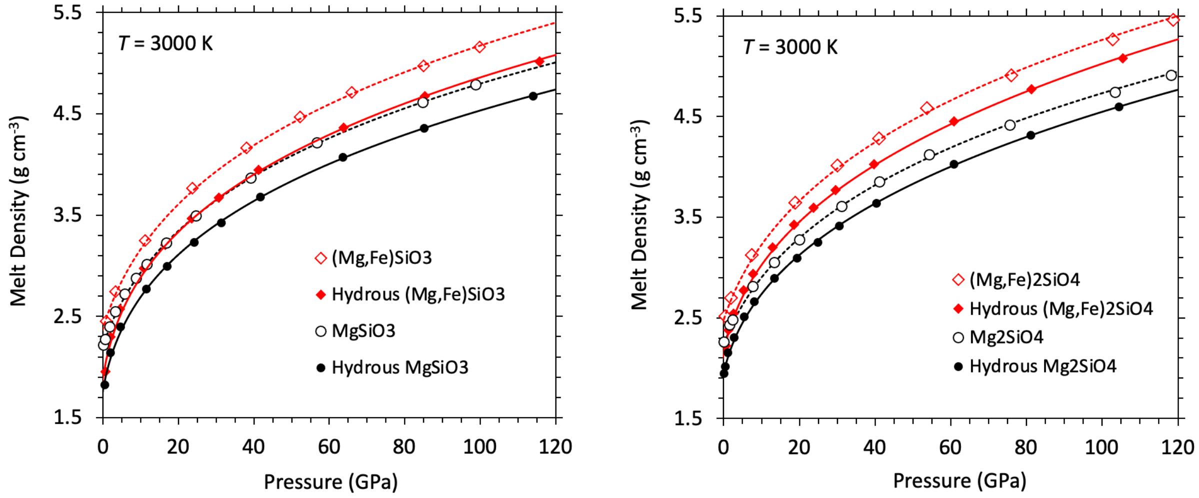


**Supplementary figure S3.** Diffusivity of hydrogen (open circles) and oxygen (open diamonds) in hydrous $MgSiO_3$ (8.2 wt% water) and $Mg_2SiO_4$ (6.0 wt% water) melts as a function of pressure at 2000, 3000, and 4000 K. The results for the corresponding iron-bearing phases are shown by small solid symbols. Also shown are the diffusivity results for $Mg_2SiO_4$ +11.4 wt% water, H: asterisks and O: pluses. The straight lines represent the Arrhenius trends for iron-free cases. The errors in the calculated diffusion coefficients are typically within 20%.

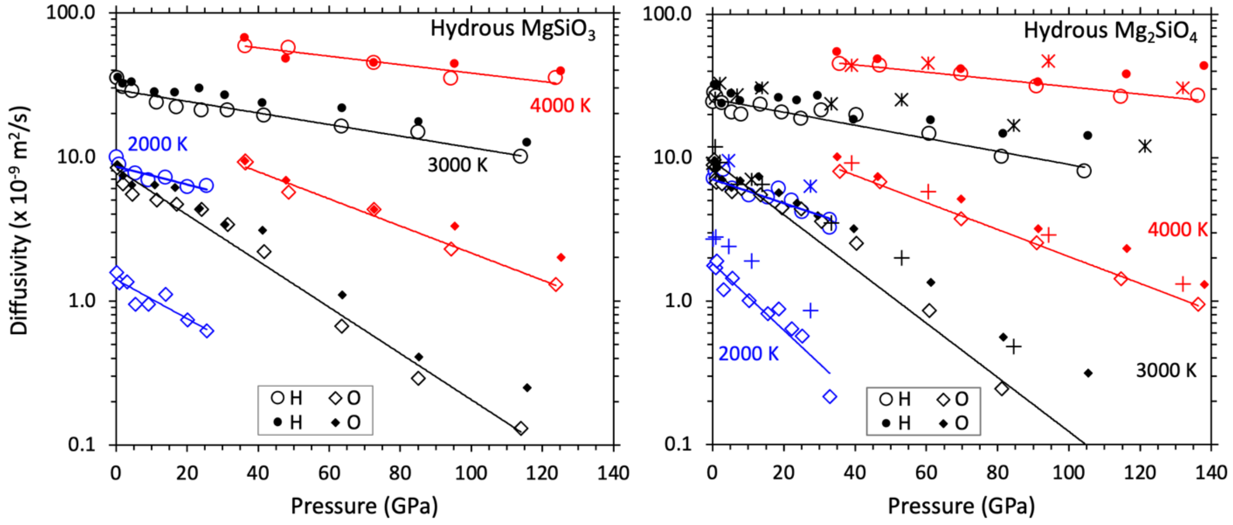

**Supplementary figure S4.** RDF matrix (symmetric) plot showing all radial distribution functions (like and unlike atom pairs) of hydrous $Mg_{0.75}Fe_{0.25}SiO_3$ melt (8.2 wt.% water) at three different conditions: 0 GPa and 2000 K (black, blue and red curves), 23.5 GPa and 3000 K (green curves), and 125.2 GPa and 4000 K (brown curves). The vertical lines mark the first peak and the minimum after the first peak. The H-Fe RDF shows a shoulder around 1.6 Å with its amplitude increasing with compression. This feature represents a direct bonding between hydrogen and iron. A peak appears around 0.7 Å in the H-H RDF (corresponding to molecular hydrogen) at zero pressure but it almost disappears at higher pressures.

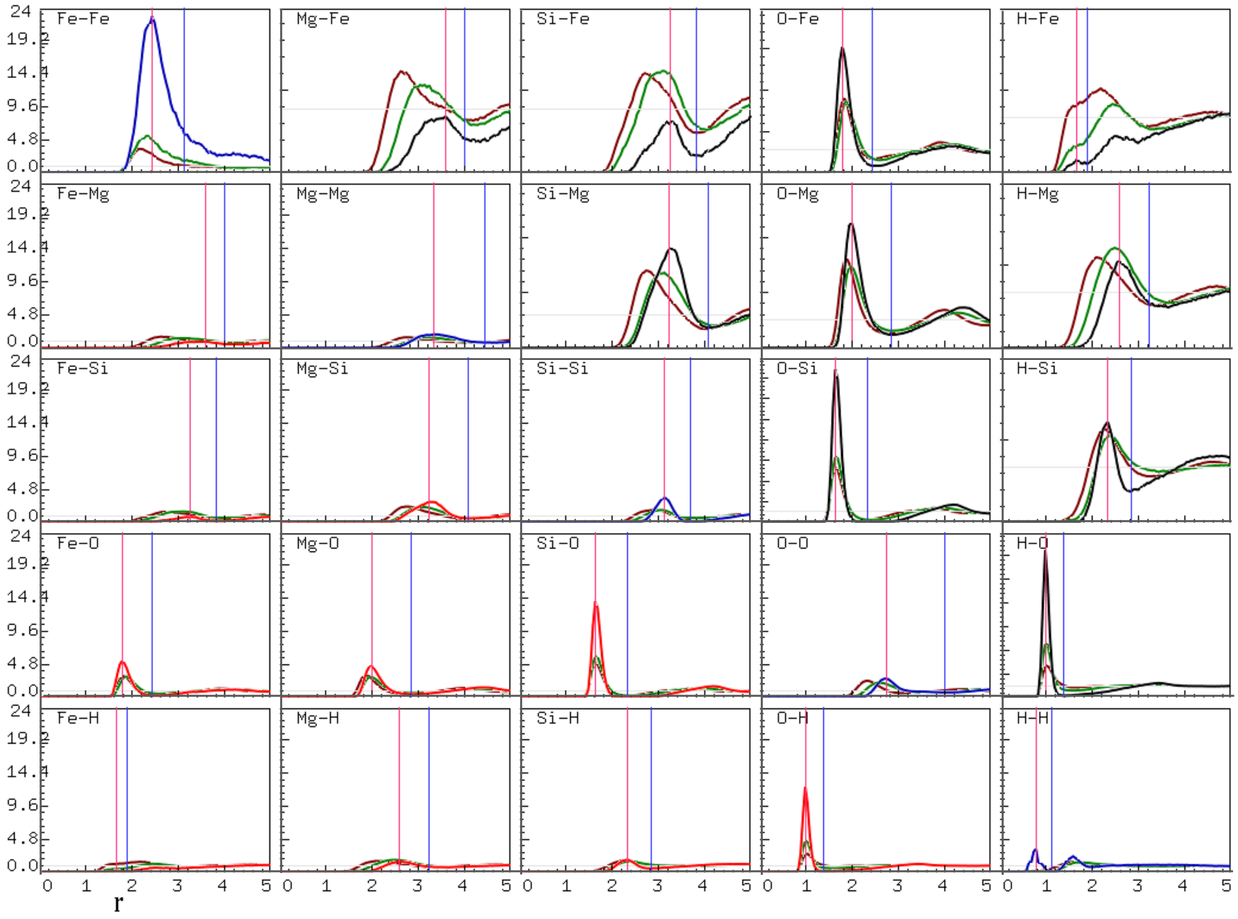

**Supplementary figure S5.** The H-O and H-H radial distribution functions of hydrous $MgSiO_3$ melt (solid curves) compared with those of pure water (dashed curves) at three pressure-temperature conditions as shown. The H-O RDF shows a clear first peak at all conditions, signifying direct bonding between oxygen and hydrogen in both silicate melt and water. The peak position is almost the same between water and melt, also being insensitive to pressure. The right side of the peak slightly extends to large distance and the minimum after the peak becomes non-zero with compression, indicating the absence of a clear first coordination shell. A weak shoulder (peak) in the H-H function around 0.7 Å detected in some simulation runs of hydrous melt implies the presence of metastable $H_2$ molecule. At low pressure, liquid water shows a clear peak at distance ~1.5 Å corresponding to the H-H distance within $H_2O$ molecule. The peak becomes shorter and boarder with further compression.

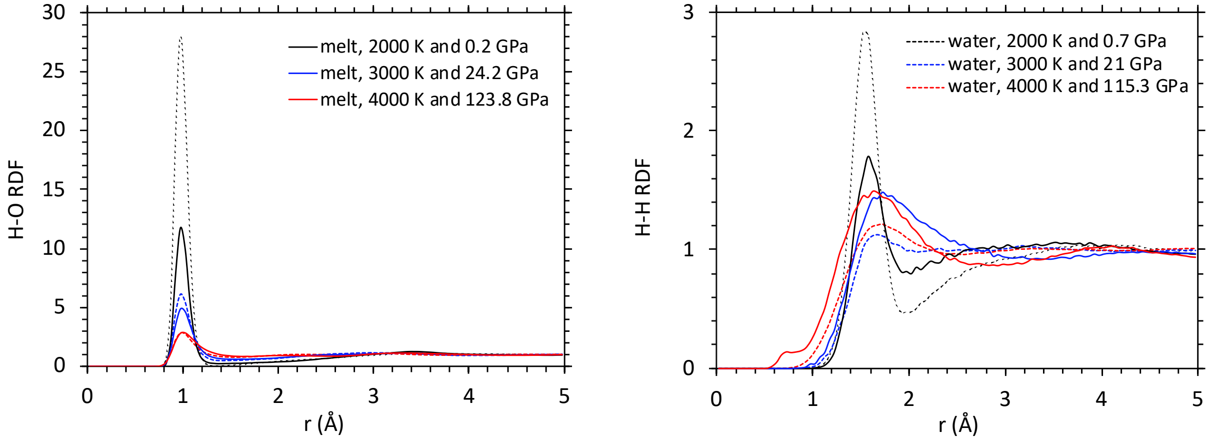


**Supplementary figure S6**. The H-O and O-H coordination distributions of hydrous $MgSiO_3$ (8.2 wt% water) and $Mg_2SiO_4$ (6.0 wt% water) melts shown by circles and diamonds compared to those of pure water (squares and asterisks) as a function of pressure at 2000, 3000 and 4000 K. The results for the corresponding iron-bearing melts are shown by small solid circles and diamonds. The numbers in boxes denote the coordination states (1: one-fold, 2: two-fold, 3: three-fold, 4: four-fold, and 5: five-fold). Also shown are the results for hydrous $Mg_2SiO_4$ with 11.4 wt% water (pluses).

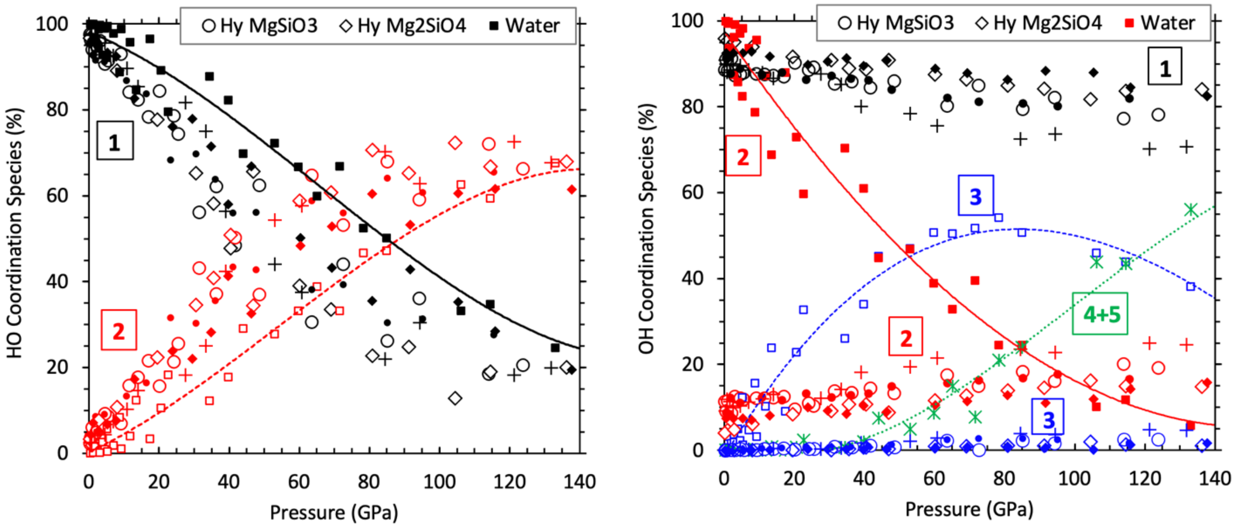

**Supplementary figure S7.** Comparison of the partial molar volume of water in silicate melts at 3000 K between the generalized gradient approximation (GGA) and local density approximation (LDA)-based calculations. The results for hydrous $MgSiO_3$ (8.2 wt% water) and $Mg_2SiO_4$ (6.0 wt% water) with GGA used in this study are systematically larger than the previous results for hydrous $MgSiO_3$ (10.7 wt% water)[5,6], hydrous model basalt (5.0 wt% water)[7], and hydrous silica (8.25 wt% water)[8]. Also note that the molar volume of pure water based on GGA (this study) is larger than the LDA volume[7] and tends to better agree with the experimental data (Fig. 2).

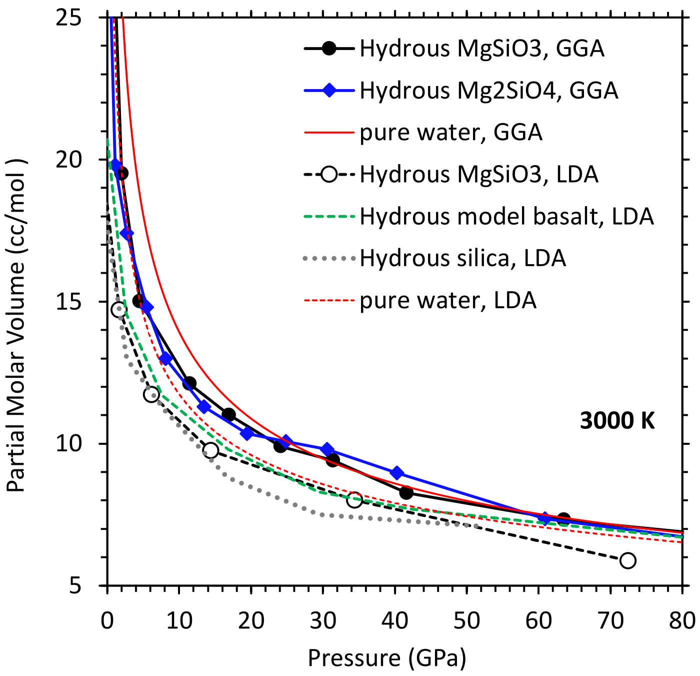


**Supplementary figure S8.** Density contrast between dry and hydrous melts for iron-free compositions (left) at 2000 K (green symbols), 3000 K (black symbols) and 4000 K (red symbols) and iron-bearing composition (right) at 3000 K (black symbols) and 4000 K (red symbols). The curves represent the model results using:

$(\rho - \rho_0)/\rho_0 = (-0.548 + 0.058\ln(P))(x_m/100)$ for iron-free melts (left)

$(\rho - \rho_0)/\rho_0 = (-0.564 + 0.054\ln(P))(x_m/100)$ for iron-bearing melts (right).

The model (Eq. 4) given in the main text was derived by considering all results together.

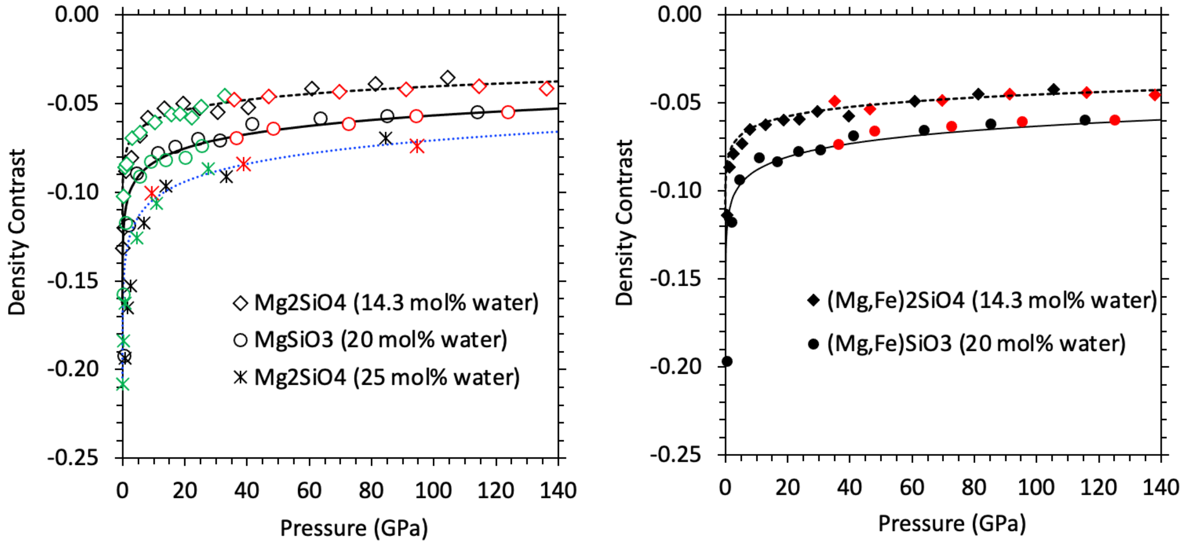

**Supplementary figure S9.** Hydrogen-induced electrical conductivity ($\sigma$) of hydrous $MgSiO_3$ (20 mol% water) and $Mg_2SiO_4$ (14.3 mol% water) melts as a function of pressure at different temperatures. Small solid symbols represent the results for the corresponding iron-bearing melts at 3000 and 4000 K. The averages taken between the iron-free and iron-bearing compositions are also shown (lines).

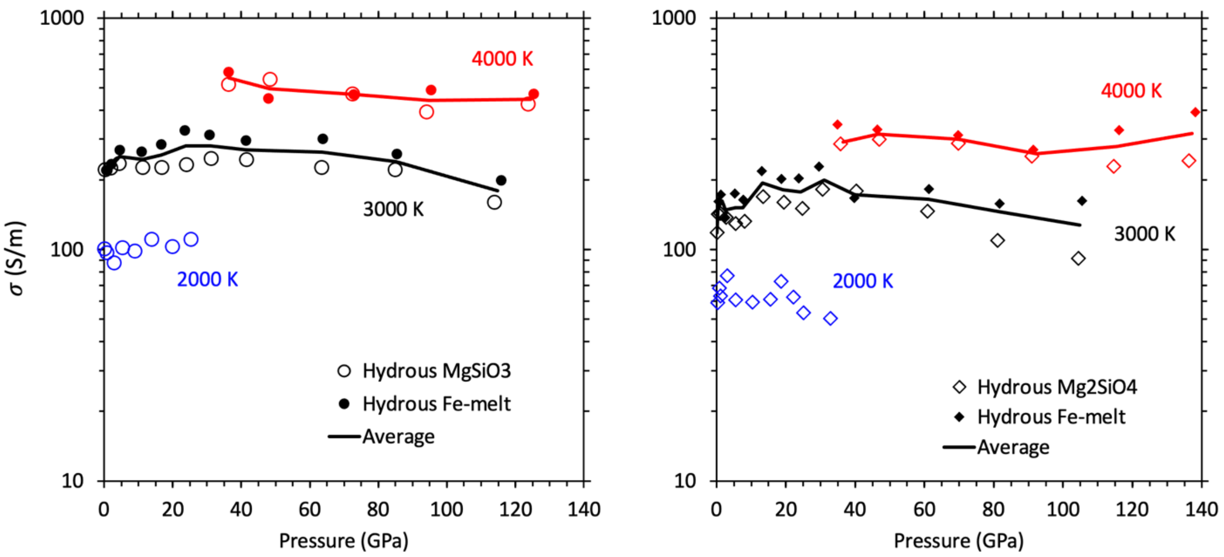


## References


1. Jing, Z. and Karato, S. A new approach to the equation of state of silicate melts: an application of the theory of hard sphere mixtures. *Geochim. Cosmochim. Acta* 75, 6780–6802 (2011).
2. Jing, Z. and Karato, S. Effect of $H_2O$ on the density of silicate melts at high pressures: Static experiments and the application of a modified hard-sphere model of equation of state. *Geochim. Cosmochim. Acta* 85, 357-372 (2012).
3. Hashin, Z. & Shtrikman, S. A variational approach to the theory of the effective magnetic permeability of multiphase materials. *J. Appl. Phys.* 33, 3125–31 (1962).
4. Waff, H. S. Theoretical considerations of electrical conductivity in a partially molten mantle and implications for geothermometry. *J. Geophys. Res.* 79, 4003–4010 (1974)
5. Mookherjee, M., Stixrude, L. & Karki, B. B. Hydrous silicate melt at high pressure. *Nature* 452, 983-986 (2008).
6. Karki, B. B., Bhattarai, D., Mookherjee, M. & Stixrude L., Visualization-based analysis of structural and dynamical properties of simulated hydrous silicate melt. *Phys. Chem. Mineral.* 37, 103-117 (2010).
7. Bajgain, S., Ghosh, D.B. & Karki, B.B. Structure and density of basaltic melts at mantle conditions from first-principles simulations. *Nature Comm.* 6, 8578 (2015).
8. Karki, B. B. & Stixrude, L. First-principles study of enhancement of transport properties of silica melt by water. *Phys. Rev. Lett.* 104, 744-751 (2010).
